# Increased lead concentrations in the hairs of radiographers in general hospitals

**DOI:** 10.1038/s41598-020-80721-3

**Published:** 2021-01-08

**Authors:** Mao-Chin Hung, Peter Chang

**Affiliations:** 1 Department of Medical Imaging and Radiological Sciences, Tzu Chi University of Science and Technology, 880, Sec. 2, Chien-Kuo Rd., Hualien, Taiwan; 2 Tufts University, School of Medicine, Boston, USA; 3 Chung-Shan Medical University, School of Medicine, Taichung, Taiwan

**Keywords:** Health occupations, Risk factors

## Abstract

This study investigated lead concentrations in the hairs of radiographers working in the radiological departments of general hospitals that used lead shielding for radiation protection. We collected scalp hair samples from 32 radiographers working in four radiology departments with lead shielding and 18 administration personnel in the same hospitals without lead shielding. Samples were analyzed for lead concentrations by inductively coupled plasma mass spectrometry. As a result, lead concentrations in the hairs of the radiological technologists were significantly higher than those in the administration staffs (0.72 ± 0.51 vs. 0.19 ± 0.27 μg/g, P < 0.001). The hair lead concentrations were positively and significantly associated with environmental lead concentrations (r = 0.6, P = 0.001), but not associated with age, working duration, and gender distribution.

## Introduction

Lead has very extensive applications, including lead storage batteries, lead bullets, and lead ingot manufacturing. The main causes of human exposure to lead at workplaces are by inhalation of lead dust and smoke present in the air^1^. Lead dust generally refers to solid particles of various sizes produced during processing, grinding, or heating of lead-based materials. Specifically, lead oxide is small particle of approximately 100 nm and easier to enter pulmonary alveoli of the exposed workers. In addition, lead can enter the body through smoking and oral ingestion of residues adhering to clothing, food, and hands^2^.

Workers with prolonged exposure to lead could develop ischemic heart disease, cerebrovascular disease, angina pectoris, or cerebral infarction^3^. In addition, health hazards caused by lead exposure including adverse effects on respiratory, digestive, reproductive, urinary and neurological systems such as neurodevelopment delay, Alzheimer’s disease (AD), Parkinson’s disease (PD), amyotrophic lateral sclerosis (ALS), attention deficit/hyperactivity disorder (ADHD), etc.^4–7^. Lead can be absorbed through the gastrointestinal tract, respiratory tract, or skin and then accumulated in various tissues^8^. The dietary lead concentrations and lead concentrations in the blood, hairs, livers, kidneys, muscles, and urine were highly positively correlated^9^.

Relevant studies have shown that determination of lead in the hairs facilitates diagnosis of lead poisoning. The hair lead measurement has been well documented and is the simplest and most effective method for screening lead poisoning and monitoring environmental lead pollution^10,11^. Due to slow growth, elements measured in the hairs represent a protracted accumulation of the elemental exposure from food, drink, and air. The hair sample analysis thus has an advantage over blood and urine sample analyses, which reflect the current and recent exposure^12^.

Because lead has a high atomic number, i.e. 82, it can produce massive photoelectric effects with x-ray and thus attenuate x-ray energy effectively. Therefore, lead is the most popular shielding material against x-ray radiation in most medical facilities^13^. In addition, the radiological departments are usually in the confined spaces with limited ventilation in the hospitals; the ambient lead concentrations in the x-ray rooms could easily cumulate, thereby increasing lead exposure of the radiological practitioners. This study aims to evaluate the accumulated lead levels of radiographers in general hospitals.

## Results

The lead concentrations in the hairs of radiographers were significantly higher than those of the administration staffs (0.72 ± 0.51 vs. 0.19 ± 0.27 μg/g, P < 0.001), while age, working duration, and gender distribution were not shown significant difference between the two groups (33.9 ± 8.6 vs. 35.2 ± 11.9 years of age; 8.4 ± 8.3 vs. 5.4 ± 5.4 years of working duration, as shown in Table 1).

**Table 1:** Comparisons between the radiographers and administration staffs.

	Radiographers	Administration staffs	D (95%CI)	t	p
Environmental lead concentration (mg/g)	3.31 ± 2.86 (0.23–8.92)	ND^a^	**–**	**–**	**–**
Hair lead concentration (μg/g)	0.72 ± 0.51 (0.08–1.86)	0.19 ± 0.27 (0.00–0.81)	0.53 (0.27, 0.79)	4.05	< 0.001
Age (year)	33.90 ± 8.57 (20–54)	35.22 ± 11.94 (21–58)	− 1.32 (− 7.18, 4.55)	− 0.45	0.134
**Gender (n, %)**					
Male	9 (28.1%)	8 (44.4%)			
Female	23 (71.9%)	10 (55.6%)	0.16 (− 0.12, 0.45)	1.16	0.070
Working duration (year)	8.38 ± 8.34 (1–27)	5.41 ± 5.36 (1–18)	2.97 (− 1.43, 7.37)	1.36	0.067

^a^Not detected or below detection limit.

The lead concentrations of the study population were further analyzed by multiple linear regression, with the lead concentration (µg/g) as a dependent variable and associated factors including age (year), gender (male/female), working duration (year) and environmental lead concentrations (mg/g) as independent variables. As a result, lead concentrations in the hairs of study population were positively and significantly associated with environmental lead exposure (r = 0.599, P = 0.001), but not associated with age, working duration, and gender distribution as shown in Table 2.

**Table 2:** The factors associated with lead concentrations in the hairs of study population.

	B (95%CI)	β	p
Constant	− 0.215 (− 0.972, 0.542)		0.567
Age (year)	0.009 (− 0.010, 0.027)	0.194	0.335
Gender (M = 1; F = 2)	0.095 (− 0.185, 0.376)	0.103	0.493
Working duration (year)	− 0.008 (− 0.038, 0.021)	− 0.110	0.568
Environmental lead concentration (mg/g)	0.155 (0.072, 0.238)	0.599	0.001
R^2^ for all factors = 35.6%			

## Discussion

The average hair growth rate is approximately 400 μm/24 h for Asian^14^, i.e. 1 cm increment per month, so hair samples including hair root less than 12 cm were collected to ensure that lead accumulation within 1 year of radiological working period and rule out possible lead accumulation or dilution beyond 1 year working period.

Any possible lead exposure at the residences and dietaries of all study subjects was ruled out, so higher lead concentrations in the hairs of radiographers may be attributed to potential lead exposure in the radiological departments. Additionally, the mean lead concentration in the hairs of radiographers (0.72 ± 0.51 μg/g) was comparable with that of workers employed in lead-related manufacturing plants (0.91 ± 0.22 μg/g)^15^. Noticeably, lead exposure on the workers could have adverse effects on their health even if their lead levels were within occupational health safety standards^16^. Therefore, the lead exposure was regard as no low limit in terms of health hazard and the lead concentration was recommended to be as low as possible.

A study on lead smelter workers found that lead was originated both from ingestion and environmental exposure; however, direct deposition from the environment was a more important source of lead in hairs^17^. Burns et al. found that 63% of lead aprons had detectable surface lead that was associated with visual appearance, type of shield, and storage method. Lead-containing shields are a newly identified, potentially widespread source of lead exposure in the health industry^18^. Additionally, lead sheets are installed within plasterboards fixed to the walls for shielding radiation in the departments of radiology, cardiology, vascular surgery, orthopedic surgery, neurosurgery, and dentistry^19^. We measured the levels of lead on wipes taken from fixed surfaces within the x-ray rooms of the study hospitals. The levels of lead ranged from 0.23 to 8.92 mg/g, and 3.31 mg/g on average. We speculate that the lead shielding materials disintegrate over time and the lead dusts escape the capillary pores of plasterboards or cracks of aprons and enter the x-ray room environments. Therefore, environmental lead accumulated in the hairs of radiological professionals during working period.

Lead sheet has been frequently and worldwide used to shield radiation in the department of radiology due to its high barrier property of radiation. However, the potential hazards of lead are ignored. This study found for the first time that increased lead concentrations in the hairs of radiographers using lead aprons for radiation protection and working in the space which installed lead shielding. Therefore, ambient lead monitoring, indoor ventilation, or replacements of lead-free shielding^20^ are recommended for avoiding such occupational hazards.

## Conclusions

Significantly increased lead concentrations were found in the hairs of radiographers working in the radiological departments of general hospitals that used lead shielding for radiation protection. Additionally, the hair lead concentrations were positively and significantly associated with environmental lead concentrations.

## Methods

### Study population

Thirty-two radiographers out of 81 radiological staffs in four general hospitals in Taiwan were recruited randomly as the exposed individuals, including 9 men and 23 women who had worked for 8 h/day and at least 1 year in the radiology departments that had installed lead for radiation shielding for more than 10 years. Eighteen administration staffs worked in the same hospital environments without lead shielding were also randomly recruited as the reference group. A questionnaire was completed by all study enrollees to rule out possible lead exposure at their residences and dietary, without hair dyed, permed, bleached, or straightened for at least 1 year before the hair collection. This study was approved by Tzu Chi Hospital Ethics Committee (approval number: IRB108-52-B). All methods were carried out in accordance with relevant guidelines and regulations (Declaration of Helsinki) and written informed consent was obtained from every participant.

### Hair lead concentration analysis

More than 200 mg of scalp hair sample was collected from every participant. The lead concentrations were analyzed using the procedure adapted from Drobyshev et al.^11^. In briefly, 1:200 (*v*/*v*) Triton X-100 solutions was used to clean hair samples, followed by acetone, and washed samples twice with deionized water. Clean samples were dried at 75 °C for 24 h in an oven, and then stored in an electronic dry cabinet at room temperature for 12 h or longer until digestion. A dried hair was digested in a microwave with 3 mL of 70% nitric acid and diluted to 10 mL with 1% (*v*/*v*) hydrochloric acid. The analyzer mixed 1 mL of the solution, 1 mL of Indium standard solution (as an internal standard) and 8 mL of 1% (*v*/*v*) hydrochloric acid to measure lead element using an inductively coupled plasma mass spectrometer (Thermo X Series II) with each sample performed in triplicate.

### Environmental lead concentration measurement

Dust wipe samples for lead were taken from the ceilings of x-ray rooms which were rarely cleaned to avoid the difference of clean frequency taking from walls. The lead concentrations of wipe samples were measured by x-ray fluorescence (XRF) spectrometry according to IEC 62321-3-1:2013. Method detect limit (MDL) for lead is 50 ppm.

### Statistical analysis

Student’s t-test was applied to analyze the differences between lead concentrations in the hair samples of the exposed and the reference groups, with statistical significant level set at P < 0.05. Multiple linear regression analysis was applied to determine the factors associated with lead concentrations in the hairs of the study population. All statistical analyses were performed using IBM SPSS Statistics 19.0 (IBM Taiwan Corp. Taipei, Taiwan).

### Sample size calculation

We were planning a study of a continuous response variable from independent control and exposed subjects. In a previous study the response within each subject group was normally distributed with standard deviation 0.5. If the true difference in the exposed and control means is 0.5, we will need to study 17 exposed subjects and 17 control subjects to be able to reject the null hypothesis that the population means of the exposed and control groups are equal with probability (power) 0.8. The Type I error probability associated with this test of this null hypothesis is 0.05^21^. Sample size calculation was performed using Power and Sample Size Calculation version 3.1.6.
